# Correction: Micheli et al. Treatment of Non-Alcoholic Steatosis: Preclinical Study of a New Nutraceutical Multitarget Formulation. *Nutrients* 2020, *12*, 1819

**DOI:** 10.3390/nu16132130

**Published:** 2024-07-03

**Authors:** Laura Micheli, Alessandra Pacini, Lorenzo Di Cesare Mannelli, Elena Trallori, Roberta D’Ambrosio, Carlo Bianchini, Pietro Lampertico, Carla Ghelardini

**Affiliations:** 1 Department of Neuroscience, Psychology, Drug Research and Child Health-Neurofarba—Pharmacology and Toxicology Section, University of Florence, 50139 Florence, Italy; laura.micheli@unifi.it (L.M.); trallorielena@gmail.com (E.T.); carla.ghelardini@unifi.it (C.G.); 2 Department of Experimental and Clinical Medicine, Anatomy and Histology Section, University of Florence, 50134 Florence, Italy; alessandra.pacini@unifi.it; 3 Foundation IRCCS Ca’ Granda Ospedale Maggiore Policlinico—Division of Gastroenterology and Hepatology—CRC “A. M. and A. Migliavacca” Center for Liver Disease, 20122 Milan, Italy; roberta.dambrosio@policlinico.mi.it (R.D.); pietro.lampertico@unimi.it (P.L.); 4 Apharm srl, 28041 Arona, Italy; carlo.bianchini@apharm.it; 5 Department of Pathophysiology and Transplantation, University of Milan, 20122 Milan, Italy

In the original publication [1], there was a mistake in Figure 8 as published. During the assembly of the panel, the conditions “HFD + AP-NHm 1 co-treatment, panel c” and “HFD + AP-NHm 2 post injury, panel f” were unintentionally represented by the wrong images. The corrected Figure 8 appears below. The authors state that the scientific conclusions are unaffected. This correction was approved by the Academic Editor. The original publication has also been updated.

## Figures and Tables

**Figure 8:**
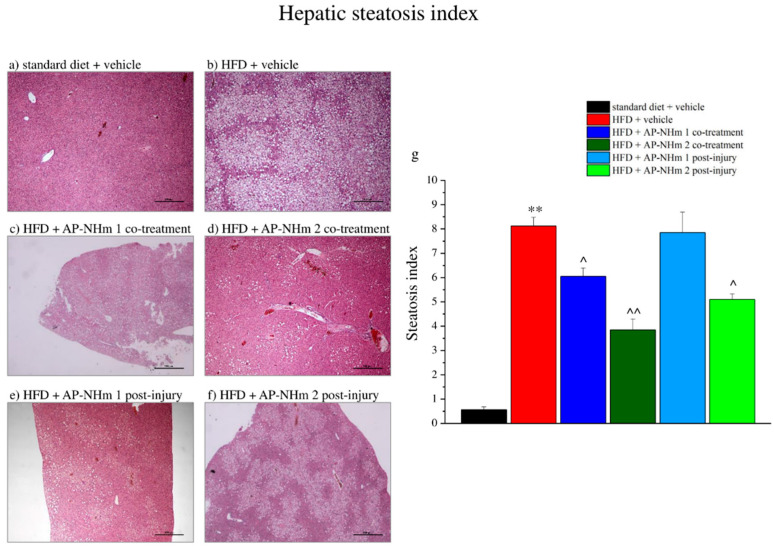
Effects of AP-NHm 1–2, in co-treatment or in post-injury treatment, on hepatic tissue and steatosis index. (**a**–**f**) Histology of tissue sections of hepatic tissue: (**a**) standard diet + vehicle, (**b**) HFD + vehicle, (**c**) HFD + AP-NHm 1 co-treatment, (**d**) HFD + AP-NHm 2 co-treatment, (**e**) HFD + AP-NHm 1 post-injury, (**f**) HFD + AP-NHm 2 post-injury; (**g**) steatosis index of each group of treatment. Each value represents the mean ± SEM of 12 mice per group. ** *p* < 0.01 vs. standard diet + vehicle, ^ *p* < 0.05 and ^^ *p* < 0.01 vs. HFD + vehicle.

## References

[1] Micheli L., Pacini A., Di Cesare Mannelli L., Trallori E., D’Ambrosio R., Bianchini C., Lampertico P., Ghelardini C. (2020). Treatment of Non-Alcoholic Steatosis: Preclinical Study of a New Nutraceutical Multitarget Formulation. Nutrients.

